# The Anti-Tumor Activity of a Neutralizing Nanobody Targeting Leptin Receptor in a Mouse Model of Melanoma

**DOI:** 10.1371/journal.pone.0089895

**Published:** 2014-02-28

**Authors:** Travis McMurphy, Run Xiao, Daniel Magee, Andrew Slater, Lennart Zabeau, Jan Tavernier, Lei Cao

**Affiliations:** 1 Department of Molecular Virology, Immunology, and Medical Genetics, The Ohio State University, Columbus, Ohio, United States of America; 2 The Comprehensive Cancer Center, The Ohio State University, Columbus, Ohio, United States of America; 3 Flanders Institute for Biotechnology, Department of Medical Protein Research, Faculty of Medicine and Health Sciences, Ghent University, Ghent, Belgium; IDI, Istituto Dermopatico dell'Immacolata, Italy

## Abstract

Environmental and genetic activation of a brain-adipocyte axis inhibits cancer progression. Leptin is the primary peripheral mediator of this anticancer effect in a mouse model of melanoma. In this study we assessed the effect of a leptin receptor antagonist on melanoma progression. Local administration of a neutralizing nanobody targeting the leptin receptor at low dose adjacent to tumor decreased tumor mass with no effects on body weight or food intake. In contrast, systemic administration of the nanobody failed to suppress tumor growth. Daily intraperitoneal injection of high-dose nanobody led to weight gain, hyperphagia, increased adiposity, hyperleptinemia, and hyperinsulinemia, and central effects mimicking leptin deficiency. The blockade of central actions of leptin by systemic delivery of nanobody may compromise its anticancer effect, underscoring the need to develop peripherally acting leptin antagonists coupled with efficient cancer-targeting delivery.

## Introduction

We recently report that living in an enriched housing environment that provides physical, social, and cognitive stimuli reduces tumor growth and increases remission in mouse models of melanoma and colon cancer [1]. Our mechanistic studies have elucidated one key mechanism underlying the anti-cancer effect of environmental enrichment (EE): the activation of a previously poorly understood neuroendocrine hypothalamic-sympathoneural-adipocyte axis (HSA). The complex environmental stimuli induce the expression of brain-derived neurotrophic factor (BDNF) in the hypothalamus and the ensuing increase in sympathetic tone to white adipose tissue. The preferential sympathetic activation of white adipose tissue suppresses leptin expression and release via action on β-adrenergic receptors leading to a robust drop of leptin level in circulation. Our pharmacological and genetic studies demonstrate that leptin is the key peripheral effector in the HSA axis mediating the anti-cancer effect of EE [1]. We have developed a molecular therapy to treat both obesity and cancer by neurosurgical delivering a recombinant adeno-associated virus (rAAV) vector in order to overexpress BDNF in the hypothalamus. This gene therapy reproduces the anti-obesity and anti-cancer effects of EE [1], [2]. In this study we investigated the effect of pharmacological blockade of leptin in the same mouse model of melanoma.

Leptin (encoded by *Ob* gene) is a pleotropic hormone primarily produced in adipose tissue. Leptin plays a crucial role in energy homeostasis by acting in the central nervous system (CNS) to increase energy expenditure and decrease feeding via a host of autonomic and neuroendocrine processes [3], [4]. In addition to its central effects in the CNS, leptin exhibits a large number of peripheral actions including modulation of immune system [5], [6], regulation of liver and muscle lipid oxidation and glucose metabolism [7]–[9], and regulation of pancreatic β-cell function [10]–[13]. Leptin mediates its effects upon binding and activation of the leptin receptor (LepR) encoded by the *Db* gene [14]. Six LepR isoforms have been characterized: a long form (LepRb or LepRlo), four short forms (LepRa, c, d, and f), and a soluble form (LepRe or sLepR) [15]. The long form LepRb is considered to possess full signaling capacity [16]. All isoforms have an identical extracellular domain consisting of two CRH (cytokine receptor homology) domains, CRH1 and CRH2, both separated by an immunoglobulin-like domain, and followed by two additional membrane-proximal fibronectin type III domains. To investigate the potential of leptin antagonists in cancer treatment, choosing a neutralizing antibody targeting the LepR instead of leptin could restrict leptin blockade to the periphery because the antibody most likely does not cross the blood-brain barrier (BBB). Zabeau *et al* generated neutralizing nanobodies targeting LepR [17]. A nanobody comprises the variable domain of the naturally occurring single-chain antibodies found in members of the *Camelidae* family [18]. The cloned variable domain is a stable polypeptide harboring the full antigen-binding capacity of the original heavy-chain antibody [19], [20]. The advantages of nanobodies compared to classical antibodies include improved tissue penetration, stability, easier genetic manipulation and production in bacteria. Nanobody 2.17 directly against the CRH2 domain of LepR blocks leptin binding to the receptor. To improve *in vivo* use, the nanobody targeting LepR was converted into a bi-specific format by fusing it to a nanobody that targets mouse serum albumin (mAlb). Binding to endogenous serum albumin greatly prolonged half-life of the bi-specific nanobody in the circulation [17]. Here we assessed the effects of the bi-specific nanobody 2.17-mAlb in the highly aggressive B16 melanoma model.

## Materials and Methods

### Mice

Male C57BL/6J mice, 6 weeks of age, were purchased from Charles River. All protocols were approved by the Institutional Animal Ethics Committees of the Ohio State University and were in accordance with NIH guidelines.

### Bispecific nanobody

The construction, production, and purification of bi-specific nanobody 2.17-mAlb were described in detail before [17].

### Melanoma implantation and nanobody treatment

We single housed mice for melanoma implantation and treatment of 2.17-mAlb. In local administration experiment, mice were shaved at the right flank. A syngeneic melanoma cell line B16 (ATCC) was subcutaneously implanted on the right flank (1×10^5^ cells per mouse). 2.17-mAlb (10 µg per mouse per injection), or PBS as a control, was injected subcutaneously adjacent to the tumor cell implantation site at day 1, 7, and 14 after tumor cell implantation. We measured the size of tumor using a caliber and calculated the tumor volume by the formula for ellipsoid (V = length×width^2^×π/6). Mice were sacrificed 18 days after tumor implantation. In systemic administration experiment, B16 cells were implanted to the right flank of mice as described above. The mice were randomized to three groups: PBS, low-dose 2.17-mAlb, and high-dose 2.17-mAlb. 2.17-mAlb or PBS was injected intraperitoneally immediately following tumor cell implantation (100 µg per mouse per injection). Low-dose 2.17-mAlb mice received 2.17-mAlb twice weekly. High-dose 2.17-mAlb mice received daily injection. Mice were sacrificed 16 days after tumor cell implantation. We dissected out the tumors from neighboring tissues and measured the weight at the time of sacrifice. In the established tumor model experiment, B16 cells were implanted to the right flank of mice as described above. On day 5 after tumor cell implantation when tumors became palpable, the mice were randomized to four groups: PBS, three doses of 2.17-mAlb treatment: 10 µg, 50 µg, and 100 µg per mouse per injection. The mice received PBS or 2.17-mAlb injections subcutaneously adjacent to the tumor implantation site on day 5, day 8, day 12 and day 15. Mice were sacrificed day 18 after tumor cell implantation.

### Body weight and food consumption

We maintained the mice on a normal 12 h/12 h light/dark cycle with food and water *ad libitum* throughout the experiment. Body weight of individual mouse was recorded twice weekly. Food consumption was recorded twice weekly as the total food consumption and represented as the average of food consumption per mouse per day.

### Serum harvest and biomarkers measurement

Blood was collected following decapitation. We prepared serum by allowing the blood to clot for 30 min on ice followed by centrifugation. Serum was at least diluted 1∶5 in serum assay diluent and assayed using DuoSet ELISA Development System (R&D Systems) for mouse leptin, adiponectin, IGF-1, and soluble leptinR. Insulin was measured using Mercodia ultrasensitive mouse insulin ELISA (ALPCO Diagnostic). Glucose was measured using QuantiChrom Glucose Assay (BioAssay Systems).

### Hypothalamic dissection

Brains were quickly isolated on ice. The hypothalamus was dissected from 2 mm-thick-coronal sections (−0.7∼−2.7 mm from bregma, 1.5 mm dorsal to the bottom of the brain, 1 mm bilateral to the midline) under a dissection scope and stored at −80°C for further analysis.

### Quantitative RT-PCR

We dissected epididymal adipose tissues and isolated total RNA using RNeasy Lipid Kit plus RNase-free DNase treatment (Qiagen). Tumor RNA and hypothalamic RNA were isolated using RNeasy mini kit plus RNase-free DNase treatment. We generated first-strand cDNA using TaqMan Reverse Transcription Reagent (Applied Biosystems) and carried out quantitative PCR using Light Cycler (Roche) with the Power SYBR Green PCR Master Mix (Applied Biosystems). We designed primers to detect the following mouse mRNAs: *Agrp*, *Cartp*, *Npy*, *Mc4r*, *Pomc*, *Insr*, *Leprb, Lep, Adipoq, Ap2, Fasn, Cpt1a, Cd31, Vegf, Kdr, Mitf, Tyrp2, and Magea4*. Primer sequences are available on request. We calibrated data to endogenous control *Actb* or *Hprt1* and quantified the relative gene expression using the equation *T_0_/R_0_* = *K*×2^(CT,R-CT,T)^. *T_0_* is the initial number of target gene mRNA copies, *R_0_* is the initial number of internal control gene mRNA copies, CT,T is the threshold cycle of the target gene, CT,R is the threshold cycle of the internal control gene and *K* is a constant.

### Cell proliferation

We cultured B16 melanoma cells (5000 cells/well in 96-well plate) with DMEM medium plus 1% mouse serum with or without 2.17-mAlb (50 µg/ml) for 3 days. Proliferation was measured using the CellTiter 96A_quesous_ One Solution Cell Proliferation Assay (Promega).

### Western blot

The dissected tumors were lysed in 100 µl RIPA buffer containing 1% proteinase inhibitor (Calbiochem 539134) by sonication. Rabbit Anti-CD31 (Abcam ab28367, 1∶300), rabbit Anti-VEGF (Abcam ab46154, 1∶1000), mouse Anti-GAPDH (Calbiochem CB1001, 1∶1000) were used in western blot analysis.

### Statistical analysis

Values are expressed as mean ± SD. We used JMP software to analyze the following: repeated measures MANOVA for food intake, weight gain, and tumor volume; one-way ANOVA for serum biomarker measurements, tumor weight and adipose tissue weight, quantitative RT-PCR data, western blot quantification.

## Results

### Local administration of a nanobody targeting LepR

**Figure 1:**
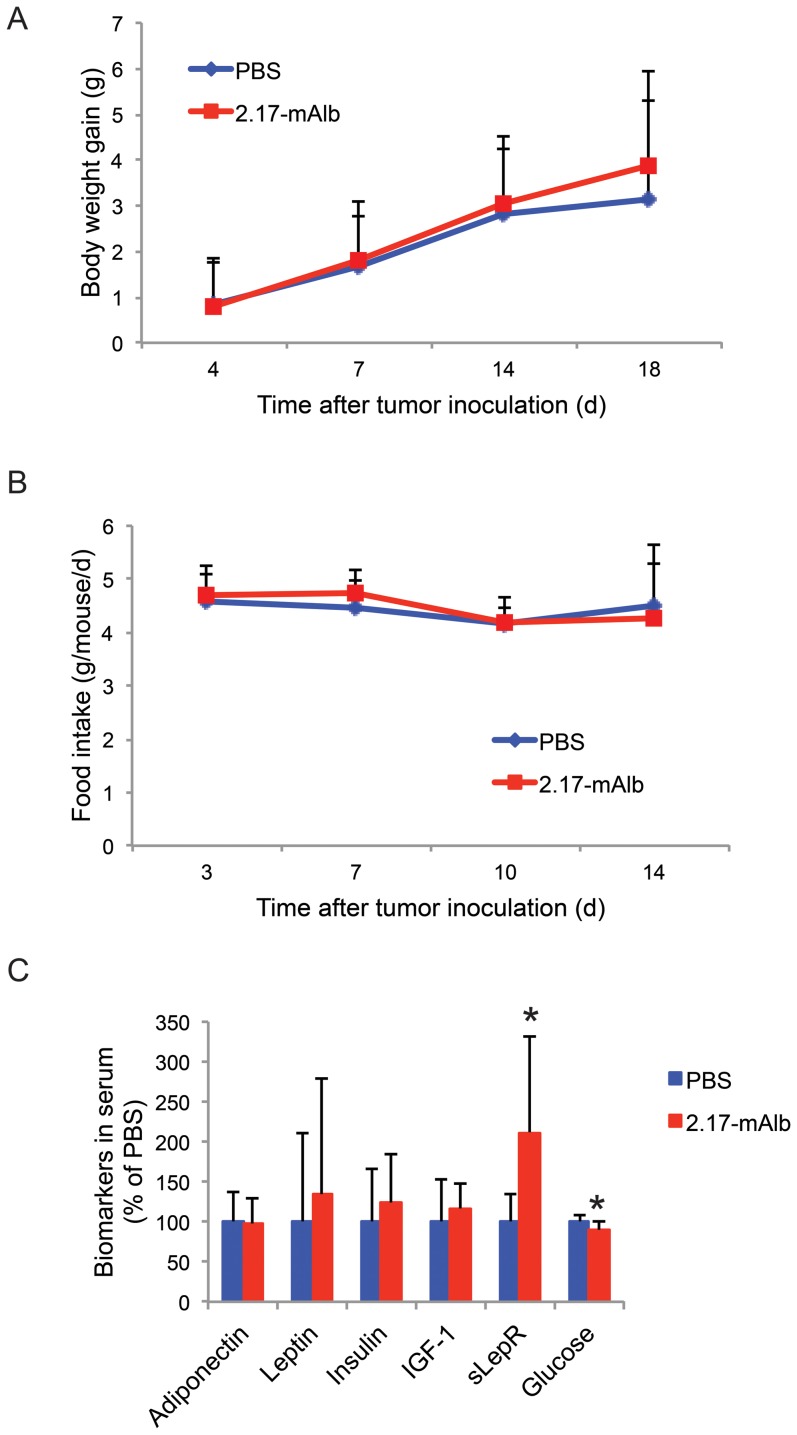
Systemic effects of local administration of 2.17-mAlb adjacent to tumor implantation site. (A) Body weight (PBS: n = 17, 2.17-mAlb: n = 23). (B) Food intake (PBS: n = 17, 2.17-mAlb: n = 23). (C) Biomarkers in serum 18 days after 3 injections of 2.17-mAlb (total dose 30 µg per mouse). n = 10 per group, * *P*<0.05. Data are means±SD.

**Figure 2:**
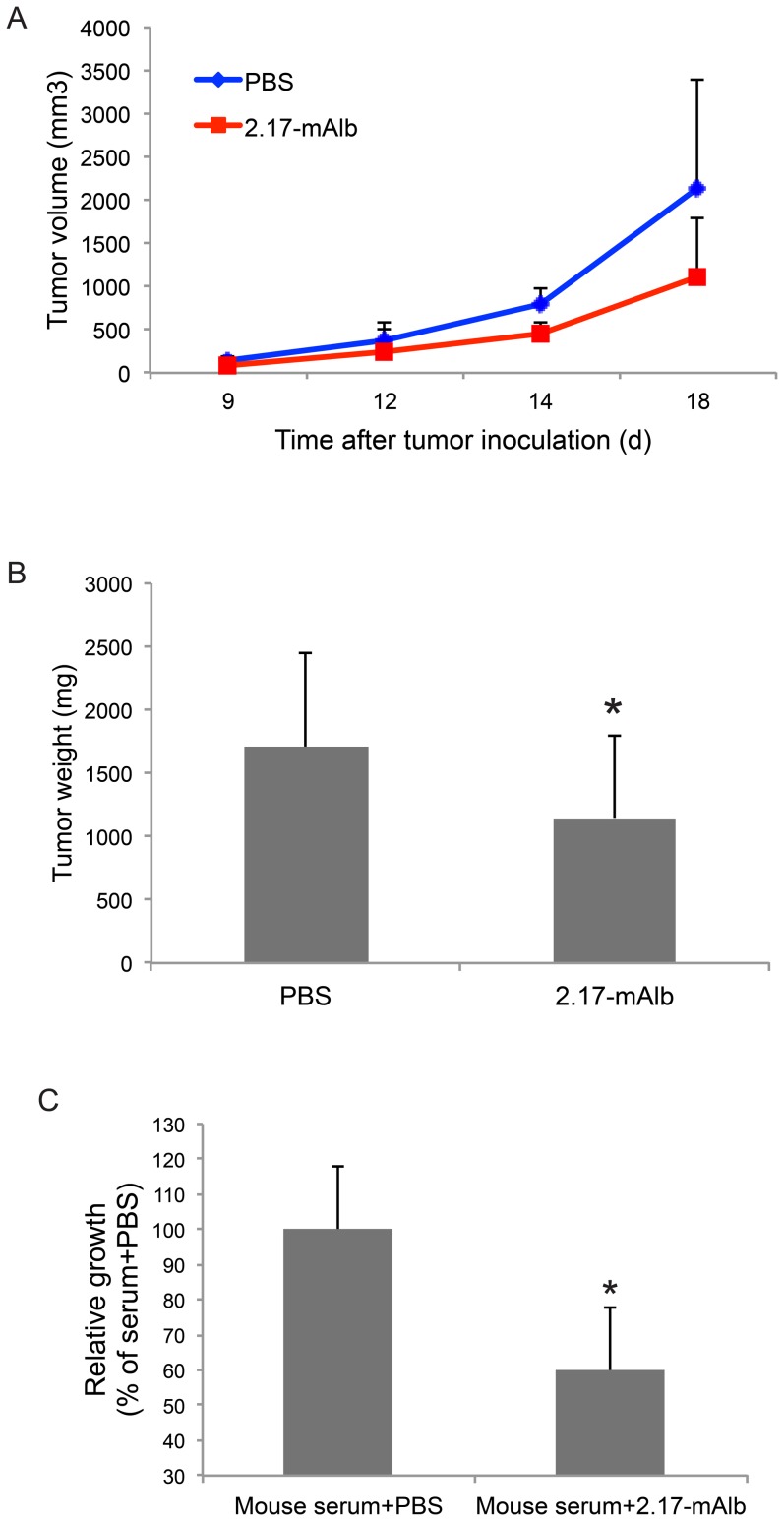
Local administration of 2.17-mAlb inhibited melanoma progression. (A) Tumor volume (*P*<0.05. PBS: n = 17, 2.17-mAlb: n = 23). (B) Tumor weight (PBS: n = 17, 2.17-mAlb: n = 23. * *P*<0.05). (C) 2.17-mAlb inhibited B16 melanoma growth *in vitro* when cultured with mouse serum (n = 4. * *P*<0.05). Data are means±SD.

**Figure 3:**
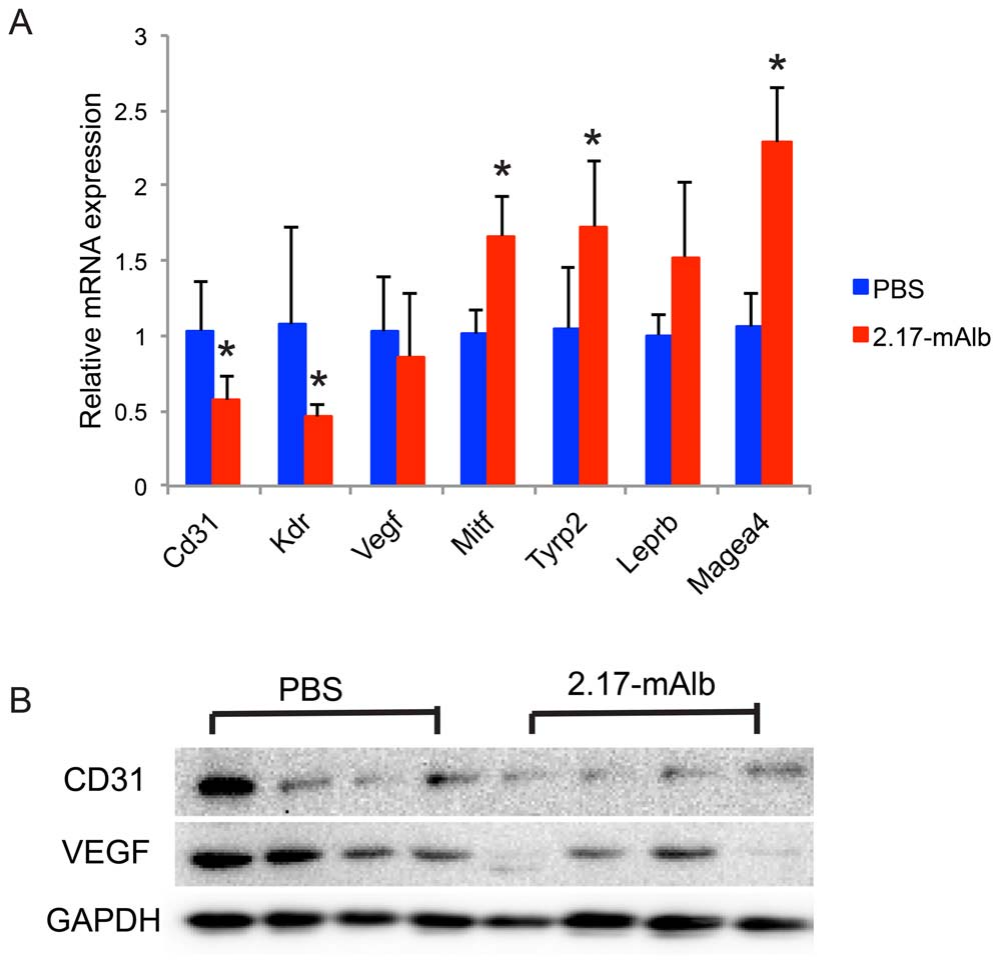
Local administration of low dose 2.17-mAlb modulated gene expression in melanoma. (A) Gene expression in tumor (n = 5 per group. * *P*<0.05). Mitf, microphthalmia-associated transcription factor; Tyrp2, tyrosinase related protein 2; Magea4, melanoma antigen family A4. Data are means±SD. (B) Western blot of tumors.

We firstly assessed the effect of nanobody 2.17-mAlb on melanoma progression when injected adjacent to the tumor implantation site. B16 melanoma cells were injected subcutaneously to the flank of male C57BL/6J mice. One day after tumor cell implantation, a low-dose of nanobody 2.17-mAlb (10 µg/mouse) or PBS was injected subcutaneously adjacent to the tumor cell implantation site. The nanobody or PBS control was injected at day 7 and day 14 at the same dose and the experiment was terminated at day 18 after tumor cell implantation. The nanobody 2.17-mAlb treatment did not affect weight gain (Fig. 1A) or food intake (Fig. 1B) indicating the absence of central effects. We observed a signature biomarker change in the serum associated with EE-induced inhibition of melanoma including decreased leptin, increased adiponectin, and decreased IGF-1 [1]. The subcutaneous administration of low-dose 2.17-mAlb had no significant effects on circulating leptin, adiponectin, or IGF-1 (Fig. 1C). Leptin inhibits insulin expression and secretion and affects β-cell mass [21]. The low-dose 2.17-mAlb had no significant effect on serum insulin while decreased blood glucose levels were observed (Fig. 1C). Interestingly, 2.17-mAlb significantly increased sLepR level in the circulation (Fig. 1C). Local administration of low-dose 2.17-mAlb (30 µg/mouse the whole course) significantly slowed the melanoma growth (Fig. 2A) and decreased melanoma mass by 33.1±7.9% (Fig. 2B). Quantitative RT-PCR was used to measure relative expression levels of transcription factors and antigens which have been associated with melanocyte differentiation and progression including microphthalmia-associated transcription factor (Mitf), silver gp100, tyrosinase, tyrosinase related protein 1, and 2 (Tyrp), as well as melanoma antigen family A2 and A4 (Mage). MITF, the transcription factor regulating the development and differentiation of melanocytes [22] was significantly elevated in 2.17-mAlb treated mice, as was TYRP-2 (Fig. 3A). MITF leads to differentiation, pigmentation and cell-cycle arrest in melanocytes. Progression of melanoma is associated with decreased differentiation and lower expression of MITF although its function may not be the same in melanoma as in normal melanocytes [23]. The increase in MITF and the genes in its pathway found in 2.17-mAlb treated animals may indicate more differentiated and less progressive tumor. Similar molecular changes were found in EE-induced inhibition of melanoma progression including increased Mitf, Maega4 and Tyrp2 (Data not shown). Leptin plays a role in modulating angiogenesis. 2.17-mAlb decreased the expression of vascular marker CD31 and the key VEGF receptor KDR that is critical to tumor angiogenesis (Fig. 3A) suggesting that the nanobody suppressed angiogenesis. Western blot showed that the VEGF protein level was significantly reduced by 60.3±12.7% (*P* = 0.042) (Fig. 3B). In an *in vitro* experiment, the expression of LepR in B16 melanoma cells was confirmed by RT-PCR. In a cell proliferation experiment, B16 melanoma cells were cultured with mouse serum. 2.17-mAlb substantially attenuated the effect of mouse serum on tumor cell proliferation (Fig. 2C). These results showed that the nanobody targeting LepR efficiently inhibited melanoma proliferation *in vitro* and tumor progression *in vivo* possibly via direct effect on cancer cell proliferation and indirect effects on tumor angiogenesis.

### Systemic administration of nanobody targeting LepR

**Figure 4:**
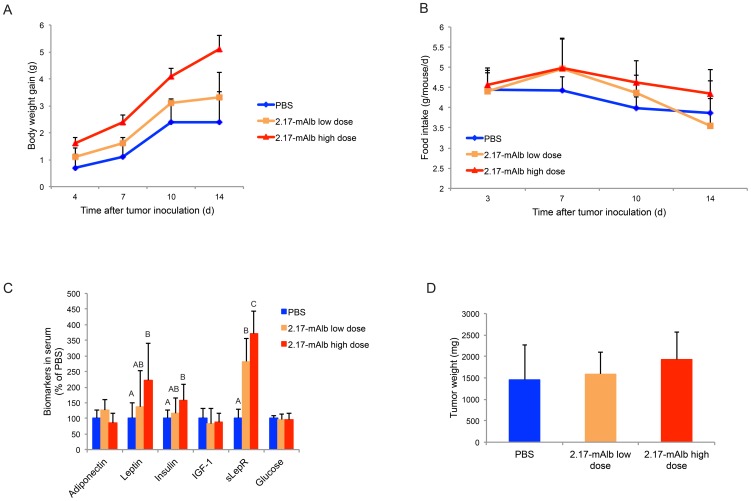
Intraperitoneal administration of 2.17-mAlb. (A) High-dose intraperitoneal administration of 2.17-mAlb accelerated weight gain (n = 10 per group, *P*<0.05 high-dose 2.17-mAlb compared to PBS and low-dose 2.17-mAlb. No significance between low-dose 2.17-mAlb and PBS). (B) High-dose 2.17-mAlb increased food intake (n = 10 per group, *P*<0.05 high-dose 2.17-mAlb compared to PBS and low-dose 2.17-mAlb. No significance between low-dose 2.17-mAlb and PBS). (C) Biomarkers in serum (n = 10 per group. Bars not connected by same letter are significantly different. (D) Tumor weight (n = 10 per group). Data are means±SD.

**Figure 5:**
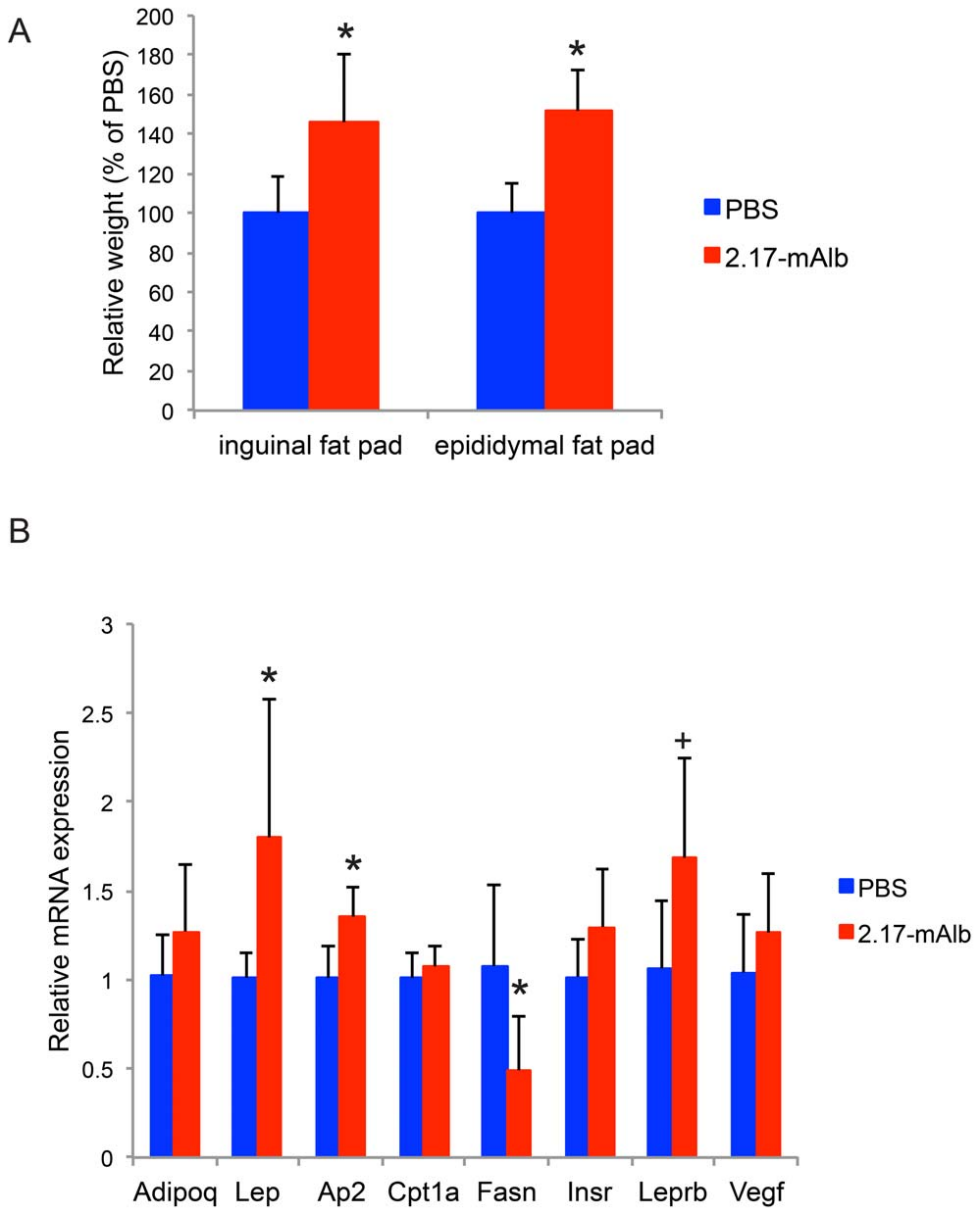
Intraperitoneal administration of high-dose 2.17-mAlb increased adiposity. (A) Subcutaneous and visceral fat pad weight (n = 10 per group. * *P*<0.05). (B) Gene expression profile of epididymal fat (n = 5 per group. * *P*<0.05, + *P* = 0.08). Data are means±SD.

**Figure 6:**
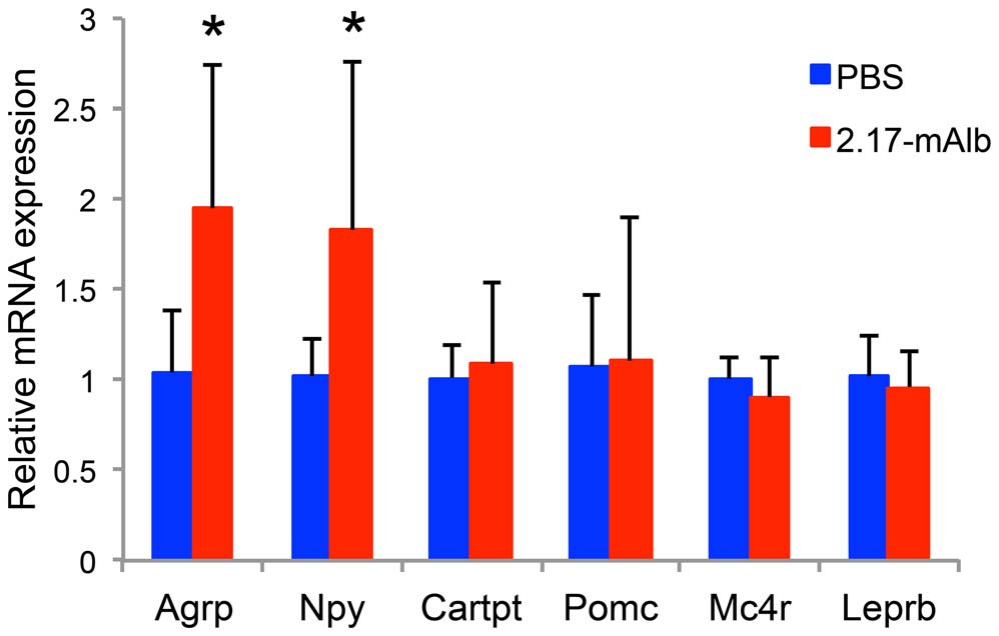
Intraperitoneal administration of high-dose 2.17-mAlb affected hypothalamic gene expression. n = 5 per group. * *P*<0.05. Data are means±SD.

We next evaluated the effects of nanobody when administrated systemically. The B16 melanoma cells were implanted to the flank of mice and the 2.17-mAlb was injected intraperitoneally (i.p. 100 µg/mouse) immediately following the tumor cell implantation. In the low-dose group, nanobody was injected twice weekly (5 injections in total). In the high-dose group, nanobody was injected daily till the end of the experiment at day 16. Intraperitoneal administration of nanobody showed dose-dependent effects on weight gain and food intake. High-dose nanobody led to accelerated weight gain (Fig. 4A) and hyperphagia (Fig. 4B) while low-dose nanobody showed no significant changes. In contrast to local administration, intraperitoneal administration of nanobody failed to inhibit melanoma growth (Fig. 4D). High-dose nanobody markedly increased the adiposity with visceral fat pad increased by 51.3±6.6% (Fig. 5A). Consistent with the increased fat mass, serum leptin level was increased in the high-dose group while adiponectin and IGF-1 were not affected (Fig. 4C). Insulin level was significantly increased in the high-dose group (Fig. 4C). The hyperleptinemia and hyperinsulinemia could compromise the anti-cancer effect of 2.17-mAlb. The sLepR level was substantially increased in both low-dose and high-dose 2.17-mAlb treated mice (Fig. 4C). The increase of sLepR was dose-dependent with high-dose i.p. 2.17-mAlb showing the largest increase while low-dose 2.17-mAlb injected locally showing the smallest change (Fig. 4C, Fig. 1C). We examined the gene expression of visceral fat by quantitative RT-PCR. High-dose 2.17-mAlb increased leptin expression in the adipose tissue (Fig. 5B). Ap2, an adipocyte differentiation marker was also increased consistent with the expansion of fat depot [24]. Leprb, the long-form leptin receptor, showed a trend of increase (Fig. 5B) probably indicating an adaptive response to the antagonism to LepR. The accelerated weight gain and hyperphagia suggested that high-dose intraperitoneal administration of 2.17-mAlb antagonized central actions of leptin. Leptin acts on two populations of neurons in the arcuate nucleus of hypothalamus, with one population expressing Pro-opiomelanocortin (POMC), the other co-expressing neuropeptide Y (NPY) and agouti-related peptide (AgRP) [25], [26]. We profiled gene expression in the hypothalamus by quantitative RT-PCR (Fig. 6). The orexigenic neuropeptides NPY and AgRP were significantly induced consistent with the increase in food intake. The anorexigenic POMC and CART prepropeptide (Cartpt) as well as the melanocortin 4 receptor (MC4R), a key pathway regulating energy balance [27], were not affected (Fig. 6).

### Nanobody targeting LepR in established tumor model

**Figure 7:**
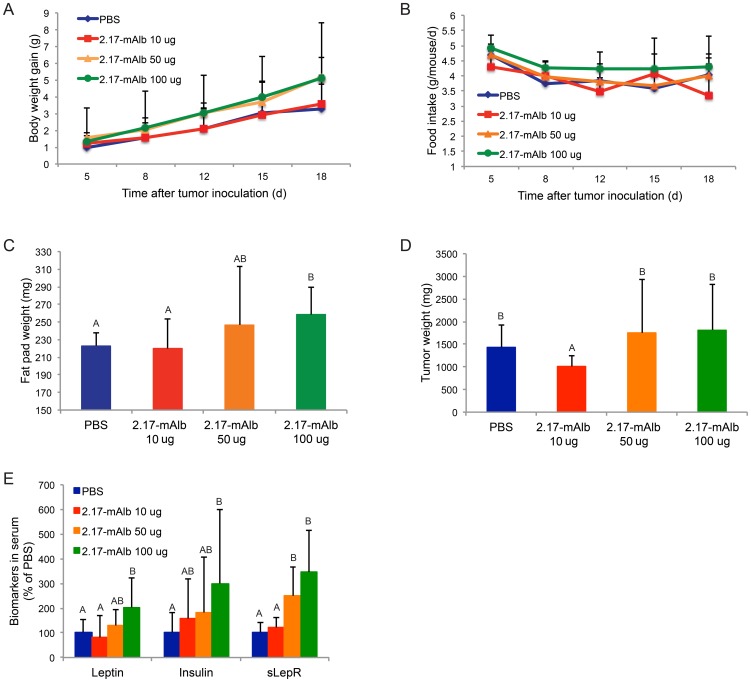
Subcutaneous administration of 2.17-mAlb in the established tumor model when treatment was delayed till palpable tumors appeared. (A) Weight gain (n = 9 per group, *P*<0.05 2.17-mAlb 100 µg compared to PBS and 2.17-mAlb 10 µg. No significance between other groups). (B) Food intake (n = 9 per group, *P*<0.05 2.17-mAlb 100 µg compared to PBS and 2.17-mAlb 10 µg. No significance between other groups). (C) Epididymal fat pad weight (n = 9 per group, bars not connected by same letter are significantly different). (D) Tumor weight (n = 9 per group, bars not connected by same letter are significantly different). (E) Biomarkers in serum (n = 9 per group, bars not connected by same letter are significantly different). Data are means±SD.

We next tested the efficacy of nanobody targeting LepR in the established melanoma model. The B16 cells were implanted to the flank of the mice. Local subcutaneous nanobody treatment was delayed to day 5 after tumor cells implantation when tumors became palpable. Three dose levels (10 µg, 50 µg, and 100 µg per mouse per injection) were used. Low dose nanobody (10 µg per injection, 40 µg the whole course) had no effects on weight gain (Fig. 7A), food intake (Fig. 7B), or adiposity (Fig. 7C). Low dose nanobody significantly decreased tumor mass even with shorter window of treatment (Fig. 7D). In contrast, subcutaneous injection of high dose nanobody failed to inhibit tumor growth (Fig. 7D). High dose nanobody treatment (s.c. 100 µg per injection, 400 µg the whole course) led to accelerated weight gain (Fig. 7A), increased food intake (Fig. 7B), increased fat pad mass (Fig. 7C), elevated leptin and insulin levels in the circulation (Fig. 7E). These changes were similar to the intraperitoneal administration of high dose nanobody (daily i.p. 100 µg per injection) although to a smaller degree (Fig. 4).

## Discussion

Leptin is not only the metabolic switch that conveys metabolic information to the brain but is also involved in multiple pathways affecting many peripheral organs as a mitogen, metabolic regulator, survival or angiogenic factor depending on the tissue type [28]. Clinical reports link elevated serum leptin levels to an increased risk of certain cancers including prostate [29], breast [30], and melanoma [31]. *In vitro* and preclinical *in vivo* data suggest that leptin acts as a mitogenic agent to promote prostate, breast, and ovarian cancer cell growth and/or enhances cancer angiogenesis and migration [32]–[34]. Thus leptin antagonists hold potential for future therapeutic use in cancer. A few anti-LepR antibodies have been generated and tested in models of heart failure [35], multiple sclerosis [36], and autoimmune encephalomyelitis [37]. An anti-rat LepR mAb reduced the growth of bone marrow leukemic cells with concomitant decrease in angiogenesis, and prolonged survival [38]. A pegylated leptin peptide antagonist (LPA) significantly inhibited breast cancer xenografts hosted by immunodeficient mice without affecting energy balance [39].

In this study we assessed the effects of a neutralizing anti-LepR nanobody in a mouse model of melanoma. Local subcutaneous administration of low-dose 2.17-mAlb significantly inhibited melanoma growth associated with decreased angiogenesis in the tumor. The absence of effects on weight and food intake suggested that the central actions of leptin were not disrupted by low-dose 2.17-mAlb although the low-dose nanobody administered adjacent to the tumor was sufficient to decrease the growth of a highly aggressive melanoma by 33%. These results further support our finding that the EE-induced anti-cancer effect was mediated, at least in part, by leptin.

The effects of high dose 2.17-mAlb are more complex. The intraperitoneal injection of 2.17-mAlb at high-dose (100 µg/mouse, daily) resulted in weight gain, hyperphagia, increased adiposity, hyperleptinemia, and hyperinsulinemia indicating efficient blockade of leptin signaling in CNS. On the other hand, low-dose 2.17-mAlb (i.p. 100 µg/mouse, twice weekly, 500 µg the whole course) showed neither significant metabolic effects nor anti-cancer effect suggesting that the antagonist availability and activity were insufficient at the respective sites of action. Therefore the overall impact of 2.17-mAlb on tumor growth was determined not only by the direct effects of LepR antagonist on tumor cells and/or other cells supporting tumor growth, but also by other systemic factors such as insulin metabolism that are regulated by leptin. In the context of cancer, insulin signaling and thus the role of leptin in the regulation of pancreatic β-cell functions are of importance. Our previous data have shown that obesity increases B16 melanoma growth in obese leptin-deficient *ob*/*ob* mice consistent with other reports [1], [40]. Prevention of the obesity by pair feeding *ob*/*ob* mice dramatically reduces tumor weight to a level significantly lower than wild-type mice of the same weight [40]. Our leptin replacement data also showed that exogenous leptin increased melanoma mass in *ob*/*ob* mice by 140% compared to pair-fed saline-infused mice with identical body weight and fat mass [1]. These data all support the role of leptin in promoting melanoma growth. The hyperinsulinemia associated with leptin deficiency in *ob*/*ob* mice may underlie the accelerated tumor growth in *ob*/*ob* mice and similarly could counteract the anti-cancer effect of 2.17-mAlb in the high-dose administration experiment. Although leptin modulates glucose metabolism via central and peripheral mechanisms, the pancreatic β-cells is a critical target of leptin actions [21]. LepRs are expressed in the β-cells and their activation directly inhibits insulin secretion. Long-term leptin deficiency inhibits insulin gene expression and β-cells mass [21]. Therefore the adverse effects on β-cells and insulin require attention for the development and application of leptin antagonists.

High dose nanobody targeting LepR blocked leptin signaling in the hypothalamus as evidenced by induction of orexigenic NPY and AgRP as well as hyperphagia and increased adiposity. There is little evidence from the literature that nanobodies are actively or passively transported across BBB [17]. The only two nanobodies known to transmigrate in an *in vitro* human BBB model and *in vivo* were generated by enrichment of a phage-display nanobody library with human cerebromicrovascular endothelial cells [41]. One explanation might be that the leptin-sensing neurons in the arcuate nucleus could make direct contact with the blood circulation [42]–[44]. Another idea is that the nanobodies targeting LepR could disrupt the transportation of leptin across BBB. In this study, we observed a robust increase of sLepR in 2.17-mAlb treated mice even when low-dose of nanobody was used. sLepR deriving from shedding of the extracellular domain is the main binding protein for leptin in the blood and modulates the bioavailability of leptin [15], [45], [46]. Experimental and clinical studies demonstrate an important role of sLepR as modulator of leptin action [47]–[50]. The regulatory mechanisms for the generation of sLepR are not well understood. A recent report suggests that lipotoxicity and apoptosis increase LepR cleavage via ADAM10 (A Disintegrin and Metalloproteinase 10) as a major protease [51]. sLepR mainly originates from short LepR isoforms [51], [52]. Leptin transport across BBB is thought to be dependent on short LepR isoforms [53]–[55]. The increase in sLepR could indicate elevated shedding of short LepR isoforms and therefore could restrain leptin transport and subsequently impair central action of leptin [56]. An alternative explanation for the increase of sLepR level in nanobody-treated mice could be that the sLepR is bound by 2.17-mAlb and thereby is retained from clearance from circulation. Therefore more research is needed to understand the regulatory mechanisms of the expression of LepR isoforms and the constitutive shedding of the extracellular domain as well as the roles of these isoforms in controlling leptin transport, bioavailability, and binding and activating signaling pathways in order to design LepR antagonists as potential therapeutics. The idea that large molecules such as nanobodies or antibodies cannot cross the BBB and therefore can restrict their actions to the periphery seems overly simplistic. Our data raise several questions in targeting leptin signaling as a treatment for cancer: how to restrict antagonizing actions to the periphery; how to prevent adverse effects such as hyperinsulinemia; how to improve bioavailability to cancer. Coupling the nanobody to the agents specifically targeting the tumor (antibody or drug conjugates) [57] may enhance the anti-cancer efficacy while prevent adverse peripheral and central effects of leptin deficiency.

In summary, we demonstrated the anti-cancer effect of a neutralizing nanobody targeting LepR in a mouse model of melanoma. Systemic administration of high dose nanobody led to blockade of central actions of leptin and may compromise the anti-cancer effect of the nanobody. These data provide insights for development of LepR antagonists as treatment for cancer.
